# EHMTI-0112. Association of age at onset of migraine with family history of migraine in children attending a pediatric headache clinic

**DOI:** 10.1186/1129-2377-15-S1-B6

**Published:** 2014-09-18

**Authors:** T Eidlitz Markus, Y Haimi-Cohen, A Zeharia, TAL Eidlitz Markus

**Affiliations:** 1 Pediatric Headache Clinic Ambulatory Day Care Hospitalization Department, Schneider Children's Medical Center of Israel Petach Tikva and Sackler Faculty of Medicine Tel Aviv University Tel Aviv Israel, Petach Tikva, Israel

## Aim

Migraine is known to cluster in families and has long been considered a strongly heritable disorder. This study sought to evaluate the relationship between age of onset of pediatric migraine and family history of migraine.

## Methods

Review of the medical files of the headache clinic of a tertiary pediatric medical center yielded 344 children with migraine for whom details on migraine in family members were available.

## Results

Mean age of the cohort was 11.69±3.49 years, and mean frequency of headache per month, 13.68±11.26. Mean age at migraine onset in patients with a negative parental history was10.48±3.39 years. Mean age at migraine onset in patients with a history of only paternal migraine was 9.29±3.64 years; with a history of only maternal migraine, 8.85±3.76 years; and with a history of migraine in both parents, 7.31±3.21 years (p=0.0001).The duration of migraine attacks (in hours) was significantly longer in patients with any family member with migraine than in those with no family history(p=0.026).

## Conclusions

Among children attending a tertiary pediatric headache clinic, migraine appears at a younger age in those with parental history of migraine relative to those with a negative family history. Anticipation may play a pathogenetic role in migraine.

No conflict of interest.

